# Enhancement of Methane Catalysis Rates in *Methylosinus trichosporium* OB3b

**DOI:** 10.3390/biom12040560

**Published:** 2022-04-09

**Authors:** Dipayan Samanta, Tanvi Govil, Priya Saxena, Venkata Gadhamshetty, Lee R. Krumholz, David R. Salem, Rajesh K. Sani

**Affiliations:** 1 Department of Chemical and Biological Engineering, South Dakota School of Mines and Technology, Rapid City, SD 57701, USA; dipayan.samanta@mines.sdsmt.edu (D.S.); tanvi.govil@mines.sdsmt.edu (T.G.); priya.saxena@mines.sdsmt.edu (P.S.); david.salem@sdsmt.edu (D.R.S.); 2 BuG ReMeDEE Consortium, South Dakota School of Mines and Technology, Rapid City, SD 57701, USA; venkata.gadhamshetty@sdsmt.edu (V.G.); krumholz@ou.edu (L.R.K.); 3 Composite and Nanocomposite Advanced Manufacturing-Biomaterials Center, Rapid City, SD 57701, USA; 4 Department of Civil and Environmental Engineering, South Dakota School of Mines and Technology, Rapid City, SD 57701, USA; 5 Department of Microbiology and Plant Biology, University of Oklahoma, Norman, OK 73019, USA

**Keywords:** active sites, docking, methanotrophs, mutation, OB3b, pMMO

## Abstract

Particulate methane monooxygenase (pMMO), a membrane-bound enzyme having three subunits (α, β, and γ) and copper-containing centers, is found in most of the methanotrophs that selectively catalyze the oxidation of methane into methanol. Active sites in the pMMO of *Methylosinus trichosporium* OB3b were determined by docking the modeled structure with ethylbenzene, toluene, 1,3-dibutadiene, and trichloroethylene. The docking energy between the modeled pMMO structure and ethylbenzene, toluene, 1,3-dibutadiene, and trichloroethylene was −5.2, −5.7, −4.2, and −3.8 kcal/mol, respectively, suggesting the existence of more than one active site within the monomeric subunits due to the presence of multiple binding sites within the pMMO monomer. The evaluation of tunnels and cavities of the active sites and the docking results showed that each active site is specific to the radius of the substrate. To increase the catalysis rates of methane in the pMMO of *M. trichosporium* OB3b, selected amino acid residues interacting at the binding site of ethylbenzene, toluene, 1,3-dibutadiene, and trichloroethylene were mutated. Based on screening the strain energy, docking energy, and physiochemical properties, five mutants were downselected, B:Leu31Ser, B:Phe96Gly, B:Phe92Thr, B:Trp106Ala, and B:Tyr110Phe, which showed the docking energy of −6.3, −6.7, −6.3, −6.5, and −6.5 kcal/mol, respectively, as compared to the wild type (−5.2 kcal/mol) with ethylbenzene. These results suggest that these five mutants would likely increase methane oxidation rates compared to wild-type pMMO.

## 1. Introduction

Methylotrophs are a diverse group of microorganisms that can utilize methane, methanol, trichloroethylene, aromatic halides, aliphatic halides, etc., as the carbon and energy sources for their growth [1]. A subset of methylotrophs, methanotrophs, can utilize only methane as the sole source of carbon for their energy and growth [2,3]. For utilizing methane, these Gram-negative methane-utilizing microbes possess a methane monooxygenase (MMO) which exists in two forms: membrane-associated particulate (pMMO) and cytoplasmic-associated soluble (sMMO) [4]. Both the pMMO and sMMO forms influence methane oxidation at their metal centers, but with distinct mechanisms for O_2_ activation and metallic complexes formation [5]. Mechanistically, differential expression of both sMMO and pMMO in methanotrophs is significantly regulated by the available copper concentration in the growth medium [6]. Hence, in our study, we unveiled whether or not the active site(s) of pMMO is(are) located within the copper center or within the vicinity of the copper center.

Except for the *Methylocella* and *Methyloferula* genera, all aerobic methanotrophs contain pMMO [7,8]. Prior studies on pMMO in the obligatory aerobic type II methanotroph, e.g., *M. trichosporium* OB3b, highlighted many interesting features related to the structure, presence of active sites, scaffold, and existence of nucleated copper sites [9,10]. In OB3b, pMMO is a heterotrimer of three subunits: α (~47 kDa), β (~24 kDa), and γ (~22 kDa) encoded by the *pmoB*, *pmoA*, and *pmoC* genes, respectively. It is commonly agreed that the expression of pMMO is highly dependent on the concentration of copper; however, the role of iron and zinc in pMMO expression remains controversial [11,12,13]. The 3.9 Å resolution of the crystal structure of the OB3b pMMO (PDB ID: 3CHX) reveals the presence of a soluble region (*pmoA*) composed of two cupredoxin-like β-barrels and a 15 α-helical transmembrane region. Furthermore, extended X-ray absorption fine structure spectroscopy shows a Cu–Cu interaction at 2.52 Å, which has also been reported for *Methylococcus capsulatus* BATH [14,15].

Over the past decade, the most debated aspect related to the OB3b pMMO has been the composition and arrangement of the metal ions in their catalytic centers. It is also unclear why the oligomerization state of pMMO is a trimer (α_3_β_3_γ_3_) although the metal centers in the three subunits (α, β, and γ) are far apart. There is further controversy regarding the active site(s), specifically whether the active site(s) is(are) in the *pmoA*, *pmoB*, or *pmoC* gene. In silico studies have shown that the active sites are present within the β-subunit [16,17]. The pMMO studied with a suicide radioactive substrate (acetylene) also showed that the active sites were present within the β-subunit [18,19]. However, the β-subunit does not contain metal centers [20]. Hence, to address this inconsistency, in the first part of this study, computational studies were performed to reidentify the active sites within the pMMO of *Methylosinus trichosporium* OB3b. Methane monooxygenases have been known to have a low affinity towards methane (due to methane’s low solubility), thus hampering methane utilization as a substrate for carbon and energy [21,22,23,24]. Protein engineering includes site-directed mutagenesis and knockdown strategies; it is quite helpful in increasing the substrate–ligand affinity, as reported in several sources [22,25,26]. Therefore, in the second part of the study, the amino acid residues in the interacting pocket sites at the α-subunit (*pmoB*) and β-subunit (*pmoA*) were studied to screen the pMMO mutants with a higher affinity towards methane. This study is necessary to identify potential strategies for increasing methane oxidation rates in *M. trichosporium* OB3b and other methylotrophs.

## 2. Materials and Methods

### 2.1. Modeling of Individual Subunits of pMMO in Methylosinus trichosporium OB3b

As discussed above, the available crystal structure of the *M. trichosporium* OB3b pMMO (PDB #3CHX) does not have good stereochemical and geometrical properties. Therefore, to model the OB3b pMMO structure, the amino acid FASTA sequences of the β-, α-, and γ-subunits of the pMMO of *M. trichosporium* OB3b were retrieved from UNIPROT with IDs Q5DV13, Q9KX50, and Q9KX51, respectively. The final sequence file was created in the PIR format by concatenating the sequences of the β-, α-, and γ-subunits. We downloaded pdball.pir.gz containing all PDB structures from the modeler website to compare the homology percentages of the available structures against the final sequence [27]. NCBI-Blast was also performed using the BLOSSUM 62 substitution matrix and the protein-specific iteration (PSI) algorithm to confirm the best homology template with good query coverage [28]. In both methods, the crystal structure of the OB3b pMMO (PDB #3CHX) showing 100% homology with the targeted structures was used as a single template to model each individual subunit of the pMMO of *M. trichosporium* OB3b. The structure was modeled using the homology modeling technique in Modeller v10.1 [29]. The selection of the best model was based on a discrete optimized protein energy (DOPE) score and a GA341 score.

The initial geometry of the best model was analyzed using Modeller v10.1 by comparing and plotting the DOPE per residue score between the template structure and the best model structure. The regions within the model structure that resulted in a less negative DOPE per residue score were corrected using the DOPE-HR loop modeling protocol in Chimera v1.14 [30]. The best model was further geometrically optimized using the CHARM22_PROT and CHARM22_CHAR force field algorithms in VEGAZZ 2.0.8 and NAMD with 100 conjugate descent steps [31]. Online software MolProbity was used to validate the model structure. In addition to the best model structure, the template structure was subjected to the Ramachandran plot analysis to compare the protein folding of each amino acid residue in the secondary structure [32]. The Ramachandran plot generated from MolProbity was used to predict the stereochemical properties of the protein. The root-mean-square deviation (RMSD) analysis using PYMOL was used to further validate the stereochemical superimposition of the atoms between the template protein and the target protein [33]. For further studies, each subunit was separated using the chain deletion technique in Chimera v1.14 and saved in individual files with the PDB extension.

### 2.2. Selecting and Designing the Substrates of pMMO

Two methane homologs, ethylbenzene and toluene, and two non-natural substrates, 1,3-dibutadiene and trichloroethylene, were chosen from the literature due to their variant radii, number of rotatable bonds, and hybridization bond patterns [34,35,36,37]. The canonical SMILES for the ethylbenzene, toluene, 1,3-dibutadiene, and trichloroethylene were noted from the PubChem database with CIDs listed in Table 1. The structures were modeled using the build editing tool in Chimera 1.14. The distances between the atoms of each substrate were estimated and formulated using the root-mean-square technique to derive the root-mean-square radii of each substrate. The geometric and stereochemical properties of the substrates are listed in Table 1, and the radii between the atoms of each substrate are shown in Figure S1. Later, the energy of the intended structures was minimized using the minimize structure option with 1000 steepest descent steps and 100 conjugate gradient steps (0.02 Å). Polar hydrogen and the Gasteiger–Marsili charges were added to all the standard and non-standard residues to count the optimized terminal charge [38]. The individual structures in the PDB format were then saved into a single PDB file using Open Babel v2.4.1 [39].

### 2.3. Interactions between the Substrates and the Wild-Type Modeled pMMO

PyRx v0.8 was used to screen the substrates by performing the docking analysis between the protein model and the set of substrates using the Vina 4.2 algorithm [40,41]. The macromolecule and the ligand structures were loaded in the workspace and converted into PDBQT files. The grid box dimensions for the x, y, and z coordinates were set at 81.78, 94.29, and 68.70 Å, respectively, so that the grid box covered the entire protein. The exhaustiveness of the system that regulates the number of independent runs, conformational flexibility of the ligands, and objective scoring function was set to 8. There were nine binding energies for each ligand–protein interaction that were confined to nine possible conformers. The best conformer interaction between the ligand and the substrate was selected based on the ligand’s highest binding energy and minimum RMSD.

### 2.4. Analysis of Hydropathy and Tunnels within the pMMO of M. trichosporium OB3b

The hydrophobicity index of the amino acid residues of the modeled structure was analyzed using the Kyle and Doolittle algorithm in Discovery Studio (DS v20, BIOVIA, Waltham, MA, USA) [42]. The hydrophobicity values were plotted in Microsoft Excel (v365), and the five-residue moving average hydrophobicity curves were analyzed using the hydrophobicity chart in DS20 to confirm the hydrophobic pockets within the modeled structure. The tunnels’ lengths and the cavities’ depths were determined using Mole v2.5.17.4.24 [43]. The interacting amino acid residues were selected, and the tunnels were evaluated by regulating the minimum bottleneck radius, pore, and length to 1.25, 0, and 0 Å, respectively. The cavities corresponding to each tunnel were found by setting the probe radius and the minimum depth to 3 and 5 Å, respectively.

### 2.5. Analysis of the Conserved Domain in M. trichosporium OB3b

The amino acid FASTA sequences of the *pmoA*, *pmoB*, and *pmoC* genes of 13 methanotrophs belonging to α-proteobacteria, γ-proteobacteria, candidate division NC 10, and *Verrucomicrobia* were retrieved from UNIPROT with UNIPROT IDs listed in Table 2. The 13 methanotrophs were selected such that each methanotroph contained the FASTA sequences of all the subunits of pMMO in the UNIPROT database. The multiple sequence alignments for each *pmoA*, *pmoB*, and *pmoC* were performed using MEGA v10, applying the UPGMA cluster method using the MUSCLE algorithm, to identify the conserved domains among these 13 methanotrophs [44]. Furthermore, to determine the evolutionary distance with regard to each individual gene, *pmoA*, *pmoB*, and *pmoC* between the 13 methanotrophs, a phylogenetic analysis for each gene was performed using the neighbor joining method and 1000 replicates (consensus bootstrap replicates) in MEGA v10. Multiple sequence alignments were performed with the BLOSSUM 62 substitution algorithm using online server Clustal Omega to evaluate the percentage similarity index between the 13 methanotrophs and individually for *pmoA*, *pmoB*, and *pmoC* [45].

### 2.6. Preparation of Mutants of the pMMO of M. trichosporium OB3b

In Discovery Studio, the docking output files and the macromolecule file with extension PDBQT were opened in the molecule window. The macromolecule was tagged to the receptor option, and the output files were set to the ligand option under the receptor–ligand interaction tab. The alkyl bond, conventional hydrogen bond, pi–sigma stacking, pi–pi stacking, and steric interactions between the ligands and the receptor amino acids were studied and noted. The Swiss PDB viewer (SPDBV 4.10) [36,46] was used to mutate the ethylbenzene-, toluene-, 1,3-dibutadiene-, and trichloroethylene-interacted amino acid residues in the modeled structure and predict the changes in the number of hydrogen bonds that account for protein stability. The PyMOL was used to determine the strain energy of the possible rotamers of each amino acid substitution. To mutate the chosen amino acids, the neighboring amino acids were chosen within 4 Å. The hydrogen bonds were displayed, and the distances were calculated. The mutate option in SPDBV 4.10 was exercised to select the intended wild-type residue within the structure and ultimately to choose the mutant residue rotamer. The strain energy of the possible rotamers was noted using the mutagenesis option available in PyMOL.

### 2.7. Interactions of the Substrates with the Mutant pMMO of M. trichosporium OB3b

To quantify the binding energies between the mutant-type modeled pMMO and the substrates, the molecular docking steps were performed using the Vina 4.2 algorithm in AutoDock 4.0 [47]. The mutant structure was processed with the addition of polar hydrogens, and Kollmann’s partial atomic charge (gasteiger charge) to minimize the energy of the structure. The protein structure with minimized energy was then saved in PDBQ file format. The binding energies between the mutants and the individual substrates were observed such that the binding site of the entire mutant was covered within the grid box. After considering the maximum distance between the ligand and the protein in all the three directions, the dimensions of the grid box were set as 20 × 15 × 20 Å. The interactions were then studied and analyzed using DS v20 by incorporating the output PDBQT ligand and macromolecule files within the molecule window.

## 3. Results

### 3.1. Analysis of the Protein Data Bank Structure of the pMMO of M. trichosporium OB3b

**Figure 1:**
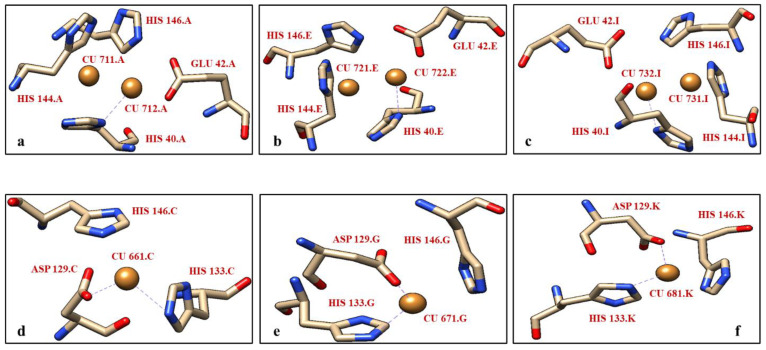
Arrangement of atoms in the pMMO of *M. trichosporium* OB3b. (**a**) Chain A having a dinuclear copper center with one free copper atom and bonded with H40, (**b**,**c**) Chain E and Chain I with the same arrangements as Chain A, (**d**) Chain C having a mononuclear copper center bonded with H133 and D120, and (**e**,**f**) Chain G and Chain K with the same arrangements as Chain C.

The crystal structure of the *M. trichosporium* OB3b pMMO retrieved from the protein databank with PDB ID #3CHX was chosen as the base model for this in silico study. The pMMO structure with X-ray diffraction resolution of 3.9 Å showed 15 peptide chains. Out of the 15 chains, nine chains were distributed between the β-subunit (A, E, I), the α-subunit (B, F, J), and the γ-subunit (C, G, K). The other six chains are unknown in terms of their function and the presence of metallic ligands. The crystal structure (#3CHX) showed the existence of dinucleated (Figure 1a–c) and mononucleated copper centers (Figure 1d–f) in the peptide chains of the α-subunit and γ-subunit of pMMO, respectively. In the α-subunit, one of the copper atoms at the dinucleated site is bonded with H40 residue while the other remains free. Interestingly, no metallic centers were present within the β-subunit. The γ-subunit, however, contains a single copper atom, bonded with H133 and D133 residues in all the three chains (C, G, K) as shown in Figure 1d–f.

In the literature, the PDB (#3RGB) structure of *Methylococcus capsulatus* BATH is characterized by the presence of trinuclear copper clusters in cellular pMMO [48]. In contrast, the crystal structure of *M. trichosporium* OB3b lacks the presence of trinuclear copper clusters. These observations indicate that the trinuclear copper center residues are not conserved among the methanotrophic genera. Hence, for the conserved domain analysis, we chose 13 methanotrophic genera for which all the genes of pMMO (*pmoA*, *pmoB*, and *pmoC*) were present within the UNIPROT database for the organism. The conserved domain analysis for *pmoC* and *pmoB* was performed among the 13 methanotrophs, namely *M. trichosporium*, *Methylocystics* sp. JTC3, *M. capsulatus* BATH, *Methylomicrobium alcaliphilum*, *Candidatus Methylomirabilis oxyfera*, *Methylacidiphilum infernorum* isolate V4, *Methylocystis* sp. strain M, *Methylocystis* sp. (strain SC2), *Methylotuvimicrobium japanense*, *Rhodococcus* sp. WAY2, *Methylovulum miyakonense*, *Methylothermus subterraneus*, and *Methylocaldum* sp. T-025. The results indicated that the neighboring amino acid residues of the mononucleated (H133, and H146) and the dinucleated (H40, E42, H142, and H144) copper center are conserved in all the methanotrophic genera. To show the evolutionary relationship between the 13 methanotrophs, phylogenetic analysis was performed each for *pmoA*, *pmoB*, and *pmoC*, individually using 1000 replicates and the neighbor joining method in MEGA v10. The dendrograms along with the scales (representing number of substitutions per site) are shown in Figure S2, each for *pmoA*, *pmoB*, and *pmoC*. For all the genes of the pMMO, *M. trichosporium* OB3b was observed to have strong resemblance (more than 90% identity) with *M. capsulatus* BATH. The multiple sequence alignment was also performed to determine the percentage similarity index between all the 13 methanotrophs with regard to each for *pmoA*, *pmoB*, and *pmoC*, individually (similarity index of the top six methanotrophs in the list is shown in Table S1). *M. trichosporium* OB3b showed a higher percentage of similarity (almost more than 50%) with *M. capsulatus* BATH for all the pMMO genes. In addition to the crystal structure, the PDBsum database showed the same sets of amino acid residues (H40, E42, H144, and H146 for *pmoB*) were conserved in *M. trichosporium* OB3b. Thus, the mononucleated copper site at *pmoC* and the dinucleated copper site at *pmoB* are conserved. Protein surfaces and their electrostatic potentials using the SPDBV (Swiss PDB Viewer) program showed that all the conserved amino acid residues of the copper centers were found to be located within the 4 Å radius of the dinucleated copper center. These copper centers may not necessarily be the pocket sites for the substrates as discussed below.

### 3.2. Modeling of the Individual Subunits of the pMMO of M. trichosporium OB3b

The modeling of pMMO against the existing PDB structure (#3CHX) was performed using the homology modeling technique with a goal to present a geometrically and stereochemically sound structure. The existing trimeric crystal structure of the pMMO of *M. trichosporium* OB3b was subjected to Ramachandran statistics using MolProbity to validate the degree of soundness of the stereochemistry of the structure. Only 48.86% of the total residues were observed to be located within the favorable region of the Ramachandran plot (shown in Figure S3). Therefore, to enhance the percentage of favorable residues, we modeled the structure of pMMO. Chains A, B, and C of the α-, β-, and γ-subunits, respectively, were modeled using Modeller v10.1. The FASTA sequences of the subunits were retrieved from UNIPROT with IDs Q9KX50, Q5DV13, and Q9KX51. The crystal structure of the whole pMMO of *M. trichosporium* OB3b retrieved from the protein databank with PDB #3CHX was chosen as the template. The template showed an identity of 100% and query coverage of 100% with the individual subunits of pMMO as found using the PSI-BLAST algorithm and the BLOSUM62 substitution matrix in NCBI-BLASTP. Modeling of the structure resulted in twenty different models with different discrete optimized protein energy (DOPE) scores, but the same GA341 score. Among the twenty different models, the structure with the minimal DOPE and GA341 scores equal to 1.0000 was taken as the best model. The DOPE and GA341 scores of the modeled structure were −89,534 and 1.0000, respectively.

**Figure 2:**
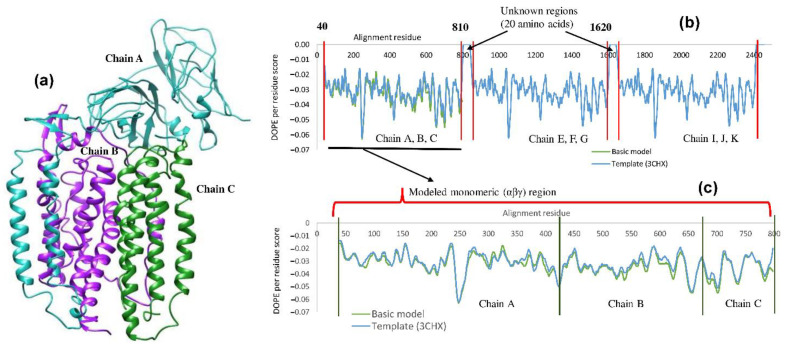
(**a**) Modeled structure of monomeric pMMO subunits (α, β, and γ) in *M. trichosporium* OB3b. Chain A is shown in light sea green color, Chain B is shown in purple color, and Chain C is shown in forest green color. (**b**) DOPE profiles of pMMO trimers. (**c**) DOPE profile of the monomeric pMMO.

The energy-minimized structure and the DOPE score per residue of the template are shown in Figure 2. The DOPE score per residue was plotted against the amino acid position for both, the template structure (#3CHX) and the best model. The DOPE score per residue shows the geometrical stability of a particular region within the secondary structure (Figure 2b). The minimum in the DOPE score per residue indicates more stability. The DOPE scores per residue of 759 amino acid residues from both the template and the model structures were statistically analyzed based on the null hypothesis principle using the f-test and the t-test in Microsoft Excel 365. The mean DOPE scores per residue of the template and the model structures in all the tests were −0.033 and −0.031, respectively, and the variances were 6.55 × 10^−5^ and 6.15 × 10^−5^, respectively. Although their mean values do not have a significant difference numerically, the null hypothesis test showed that the datasets varied significantly (Table 3). In the f-test, the value of f (1.065) is not comparable to one-tailed F critical value (1.127), whereas in the paired unequal variance t-test, the value of t (−4.2) is much less than the value of alpha chosen (0.05). The one-tail and two-tail *p*-values for the t-test between the template and the model are observed to be significantly smaller in the order of 10^−5^, suggesting a significant difference between the DOPE per residue score of the template and the model. Thus, based on the t-test (*p*-value), the null hypothesis can be rejected, which implies that significant changes occurred between the modeled and the template structures in case of energy per residue. Furthermore, the template and the model structure were superimposed to validate the potential changes in the torsion and stereochemical angles as shown in Figure S3c.

To improve the stereochemical properties of the modeled structure, the selected structure was further corrected using the loop refinement technique [49]. Loop refinements were performed in Chimera v1.14 using the Modeller v10.1 loop optimization script with manually adjusted torsion angles. A large set of trials with restraints (e.g., torsion angles, Lennard–Jones potentials, CA–CB and CA–CA bonds) led us to make the geometry of the protein better. The finalized structure modeled using a hybrid technique (homology and refinement) was further subjected to energy minimization using the CHARM22_PROT and CHARM22_CHAR force field algorithms in VEGAZZ 2.0.8 and nanoscale molecular dynamics (NAMD). The Ramachandran statistics of the modeled and template structures were performed using MolProbity (online server) and are shown in Figure S3. The percentage of residues in the favorable region of the modeled and template structures is 82.34 (4.78% in outliers) and 48.86 (22.89% in outliers), respectively, with the RMSD of 0.468 Å between the template and the protein (shown in Table 4). High percentages of residues in the favorable region and smaller RMSD values indicate sound and improved geometry of the modeled structures.

### 3.3. Interactions between the Substrates and the Modeled Structure

The reported literature has contradictory results while dealing with the active sites and metallic centers of pMMO. For example, a report stated the existence of an active site within the dicopper site of *pmoB* [20]. In contrast, methane oxidation at the monocopper site of *pmoC* was also reported, which stated no dinucleated copper center within the enzyme [50]. Further, the trimeric crystal structure (3CHX) has been reported to contain copper, iron, and zinc centers [9], whereas in our study, we did not encounter any metal sites other than copper within pmoA and pmoC. Furthermore, there is no copper site in the pmoB subunit in the structure predicted above in this study. These results pose questions of whether methane oxidation requires metal centers during their oxidation or whether other sites available within the enzyme act as the reaction centers? Therefore, interactions between the substrates and the modeled structures are deemed necessary to determine the pocket sites and claim active sites.

The previously published literature was searched to choose the substrates with different radii, number of rotatable bonds, and hybridization states of the bonds for molecular docking [51]. Since methane cannot be used as a substrate in molecular docking due to the lack of rotatable carbon-carbon bonds and flexibility, ethylbenzene and toluene were used in the study that act as methane homologs [52]. Both ethylbenzene and toluene have been reported to act as physiologically relevant substrates for monooxygenases (sMMO and pMMO) [53]. It is reported that industrial waste products such as 1,3-butadiene and trichloroethylene can be degraded using pMMO, and thus 1,3-butadiene and trichloroethylene have also been chosen as substrates [54]. The radii of 1,3-butadiene (4.34 Å) and trichloroethylene (4.89 Å) are smaller than those of the aromatic ring fused structures of ethylbenzene (8.018 Å) and toluene (7.622 Å). The number of rotatable bonds was higher in ethylbenzene among all the chosen substrates, thus leading to higher flexibility in the structure backbone of ethylbenzene. The structures of ethylbenzene, toluene, 1,3-dibutadiene, and trichloroethylene with their atom-to-atom radius are shown in Figure S1 and the geometrical and stereochemical properties are listed in Table 1.

**Figure 3:**
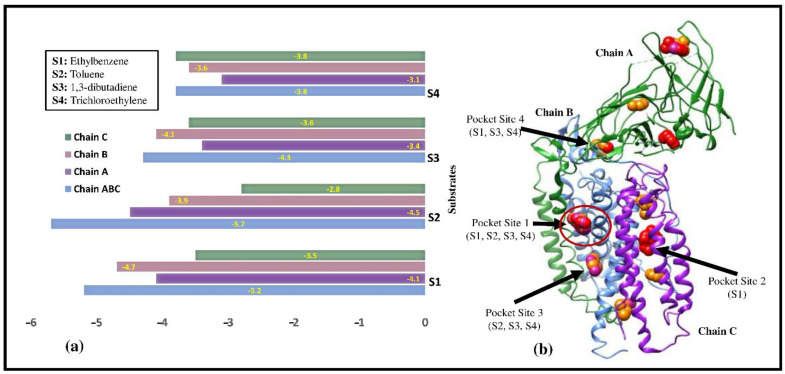
(**a**) Plot of binding energies between substrates and Chain A, Chain B, Chain C, and modeled pMMO monomer. (**b**) The surface interactions between the substrates and modeled pMMO. Chain A is shown in the light sea green color, Chain B is shown in purple, and Chain C is shown in light blue. The substrates are shown as red spheres.

The interactions were analyzed by performing the docking between the modeled structure of pMMO (Chains A, B, C) and the individual chains (A, B, or C) with ethylbenzene, toluene, 1,3-dibutadiene, and trichloroethylene. The virtual screening technique was employed using PyRx v0.8 to determine the binding energies. The binding energies of ethylbenzene, toluene, 1,3-dibutadiene, and trichloroethylene with the modeled pMMO are −5.2, −5.7 −4.3, and −3.8 kcal/mol, respectively. The binding energies between pMMO and the individual chains (A, B, and C) with ethylbenzene, toluene, 1,3-dibutadiene, and trichloroethylene are plotted in a bar chart in Figure 3a. The highest binding energy among all the substrates was observed in toluene when toluene was docked with pMMO (ABC, −5.7 kcal/mol) and chain A (−4.1 kcal/mol). However, the highest binding energies of the chains B and C were observed with ethyl benzene and trichloroethylene (−4.7 and −3.8 kcal/mol, respectively). The binding sites of ethylbenzene, toluene, 1,3-dibutadiene, and trichloroethylene are marked as pocket sites (PS) and are shown in Figure 3b. Pocket site 1 (PS1) shows the interaction of ethylbenzene, toluene, 1,3-dibutadiene, and trichloroethylene, wherein the substrates graved within the cavity formed between the β- and α-subunits. In contrast, the binding sites of other conformers of toluene are located in PS2 (the cavity between the γ- and α-subunits) and PS3 (the cavity between the β- and α-subunits). Besides, PS1, PS3, and PS4 have been observed to be the binding sites for the conformers of small molecules such as 1,3-dibutadiene and trichloroethylene. Thus, the active sites (binding sites) for the molecules with different physical dimensions are different and are distributed throughout the subunits. Furthermore, these results can be explained using the hydrophobicity analyses of the pockets as discussed below. The literature lacked such information, so we could not compare these results.

**Figure 4:**
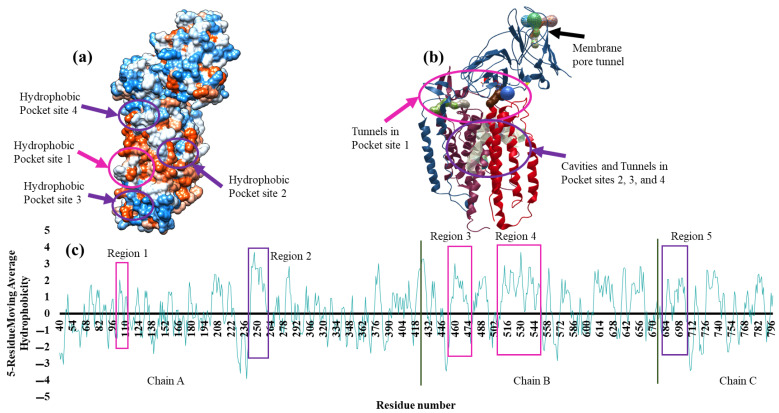
(**a**) The solvent accessible and nonpolar hydrophobic cavities in solid surface display; (**b**) The surface tunnel and cavities of modeled pMMO monomer. (**c**) 5 Residue Moving Average Hydrophobicity of monomeric pMMO subunits of *M. trichosporium* OB3b. The pocket site 1 is shown in light pink circles and rectangles, and the pocket sites 2, 3, and 4 are shown in purple circles and rectangles.

The four hydrophobic pockets picturized using the hydrophobic surface display in Chimera v1.14 are shown in Figure 4a. Figure 4b shows the cavities and tunnels analyzed using Mole 2.5.17.4.24 for PS1, PS2, PS3, and PS4. The 5-Residue Moving Average hydrophobicity was analyzed using Discovery Studio v20 and is shown in Figure 4c. The hydrophobicity index determined using the Kyle and Doolittle algorithm showed positive numbers (above the x-axis) for hydrophobic residues that govern the pocket or active sites. The pocket sites’ positions are marked in the hydrophobicity plot in Figure 4c. The depths of cavities for PS1, PS2, PS3, and PS4 are 3892, 1954, 691, and 582 Å^3^, respectively, and the length of tunnels for PS1, PS2, PS3, and PS4 are 20, 27, 23, and 17 Å, respectively. Hydrophobicity analysis showed the possibility of 15 cavities in α-subunit with a minimum bottleneck radius set to 1.25 Å. The longest tunnels among these 15 were found in 3 cavities with each of length 27 Å and average volume of 2009 Å^3^, while the average length and volume of 3 cavities among 23 cavities in β-subunit are 17 Å and 690 Å^3^, respectively. The average length and volume of 3 cavities among 18 cavities in γ-subunit are 15 Å and 558 Å^3^, respectively. The different volumetric values of the cavities suggest that α, β, and γ-subunits are equally important for the diffusional travel of molecules through the membrane and regulate the penetration of molecules to the active site. The comparative binding energy values between ethylbenzene, toluene, 1,3-dibutadiene, and trichloroethylene suggested that the lower the depth of the cavity and the greater the length of the tunnel, the less is the binding energy. Thus, in conclusion, each pocket site is highly specific to the geometry of the substrate, and there is presence of multiple active sites within the pMMO enzyme. The docking studies were performed with the holoenzyme, but no interactions were found at the vicinity of the copper center. Moreover, the holoenzyme revealed the surface accessibility of the metal center less than 5 Å^2^, which is much lower than the radii of the substrates (shown in Table 1), and therefore cannot accommodate the substrates within the metal center. The hydrophobicity analysis revealed the dominancy of hydrophilic residues at the metal center, which is significantly unlikely to be claimed as active site. Besides the non-existence of deep cavities, there were no tunnels or channels observed at the proximity of the copper centers. In conclusion, the metal centers may not be the site of methane oxidation.

**Figure 5:**
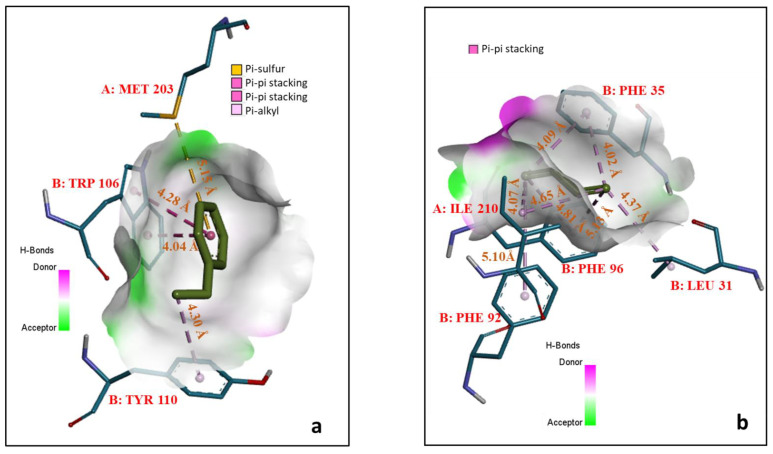

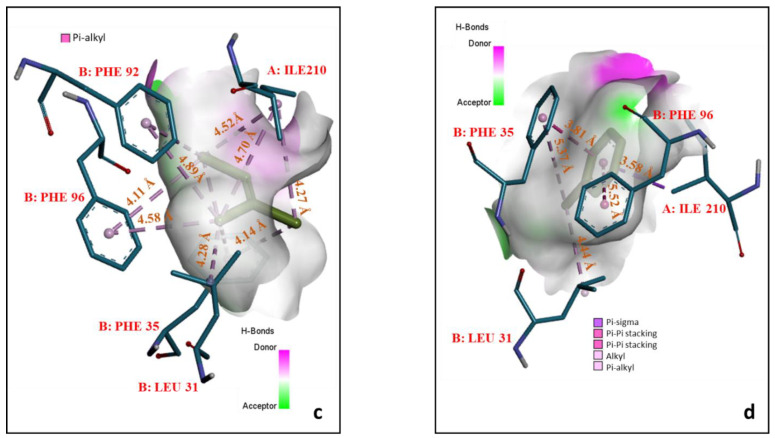
Pocket site interactions between the modeled pMMO and (**a**) ethylbenzene, (**b**) toluene (**c**) 1,3-butadiene, and (**d**) trichloroethylene. The alkyl bonds, conventional hydrogen bonds, Pi-alkyl, and Pi-sigma stacking are shown in light pink, green, dark red, and yellow, respectively.

The interaction study between the modeled pMMO and ethylbenzene, toluene, 1,3-dibutadiene, and trichloroethylene revealed the hydrophobic nonpolar amino acids that interacted with ethylbenzene, toluene, 1,3-dibutadiene, and trichloroethylene in different pocket sites, which are shown in Figure 5. When ethylbenzene, toluene, 1,3-dibutadiene, and trichloroethylene were docked with the modeled pMMO, the PS1 was observed with the interacting residues such as B:Trp106, B:Tyr110, A:Met203, B:Phe35, B:Ile210, B:Leu31, B:Phe32, and B:Phe96. The interaction between ethylbenzene and the modeled pMMO resulted in pi-sulfur, pi-pi stacking and pi-alkyl bonds (distances are less than 5 Å), with no trace of other non-covalent bonds, as shown in Figure 5a. In PS2, the occurrence of Arg (A:211, and A:207), Tyr (B:154, A:157), and Phe (B:35, A:405) was higher with more focused on pi-sigma stackings and pi-alkyl bonds when the modeled pMMO was docked with duroquinol. The docking between toluene and modeled pMMO revealed the existence of only pi-pi stacking as shown in Figure 5b. Figure 5c,d show the interactions between 1,3-dibutadiene and trichloroethylene, respectively, with the modeled pMMO. Ethylbenzene, toluene, 1,3-dibutadiene, and trichloroethylene shared the common pocket site (PS1) with conformers’ RMSD less than 1 Å.

**Figure 6:**
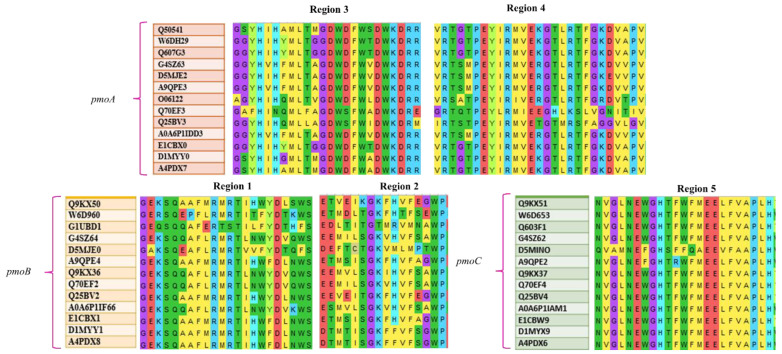
Conserved amino acid residues in *pmoA*, *pmoB*, and *pmoC* (along with UNIPROT accession number of respective species) and the correlation with the pocket regions.

The regions of the pocket sites are then validated using multiple sequence alignment in MEGA v10 to examine the chances of conservation in different methanotrophic genera as shown in Figure 6. The multiple sequence alignment performed between 13 methanotrophic genera that belong to the α-proteobacteria, γ-proteobacteria, and Verrucomicrobia shows that the conserved domain residues are hydrophobic and non-polar, and they are the same as the interacting residues. According to the hydrophobicity index of the amino acid residues surrounding the pocket sites, the residues are nonpolar and hydrophobic, which contributed to the non-electrostatic bonds between them and the substrates. However, the partial charged bonds such as convectional hydrogen bonds and electrostatic bonds are also observed on the side layers of amino acid residues surrounding the pocket sites. The electrostatic and non-electrostatic bonds together formed an intact enzyme-substrate complex [55,56]. Furthermore, no allosteric sites were recorded within the enzyme with regard to the arrangement of amino acids and binding energies observed with the pocket sites. The binding energies between the substrates and pocket sites are considerably too low to participate in allosteric inhibition [57]. Thus, the binding energy approach, hydrophobicity and multiple sequence alignment data suggest that PS1, PS2, PS3, and PS4 are active sites in monomeric pMMO. Interestingly, these pocket sites do not contain copper centers. Therefore, the role of copper center, and the mechanisms of involvement of copper during methane oxidation, remains unclear and needs to be elucidated

### 3.4. Mutation Study in Pocket Site I (PS1) in pMMO of M. trichosporium OB3b

Methane has a low solubility in nitrate minimal salt media due to low gas to liquid mass transfer [58,59]. Also, methane monooxygenases have low affinity towards methane [21]. Methane oxidation rates could be enhanced using protein engineering of pMMO to increase the affinity towards methane. We can also replace amino acids present in the active site of pMMO to increase the enzyme affinity towards methane. Furthermore, from the docking studies, the conformers of ethylbenzene, toluene, 1,3-dibutadiene, and trichloroethylene with less than 1 Å RMSD were observed to be graved within the PS1. Therefore, to carry out the mutation studies, PS1 was used where all the conformers were found to be graved within, as shown in Figure 4b. Thus, the interacting atoms B:Trp106, B:Tyr110, A:Met203, B:Phe35, B:Ile210, B:Leu31, B:Phe32, and B:Phe96 are the probable candidates that govern the methane oxidation. We hypothesize that mutation in these interacting amino acid residues will enhance the methane catalysis rates. These residues have non-bonded contacts and salt bridges with other chains present in modeled pMMO, with strong hydrogen bonding with the neighbor residues of other chains.

**Figure 7:**
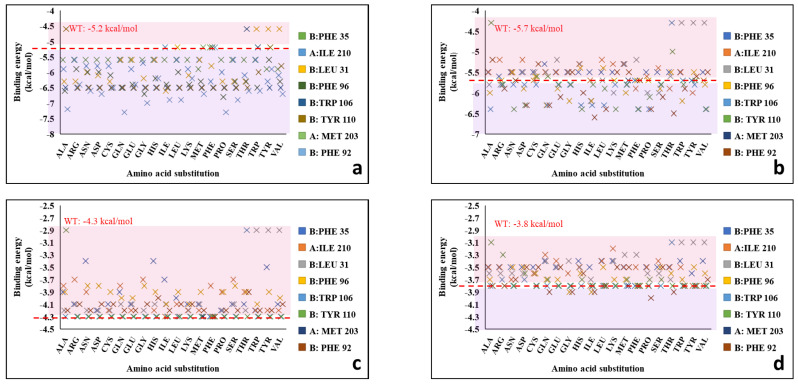
The binding energy plots between the mutants and their binding energies with (**a**) ethylbenzene, (**b**) toluene, (**c**) 1,3-butadiene, and (**d**) trichloroethylene.

Therefore, the mutations at amino acid residues B:Trp106, B:Tyr110, A:Met203, B:Phe35, B:Ile210, B:Leu31, B:Phe32, and B:Phe96were performed using SPDBV 4.10. The results were analyzed using online mutation servers MutPred and Polyphen 2.0 to confirm the variation in transmembrane properties [60,61]. Several parameters were considered that make the substitution suitable without significantly changing the physiochemical properties of the transmembrane particulate enzyme (pMMO). The strain energy was also considered since it signifies the steric hindrance caused by overlapping of Van der Waal surfaces. The less the strain energy of the rotamers of the substituted amino acid residue, the more is the stability of the structure. The strain energy of the 19 amino acids when mutated against the 8 interacting amino acids are shown in Table S2. The mutational study compared the substitution of 19 different amino acids and the most probable substitution at the metallic site of the enzyme was recorded. The mutation studies also included the consideration of hydrogen bonds of B:Trp106, B:Tyr110, A:Met203, B:Phe35, B:Ile210, B:Leu31, B:Phe32, and B:Phe96. In *M. trichosporium* OB3b with the neighbor residues along with binding affinities between the amino acid mutants and ethylbenzene, toluene, 1,3-dibutadiene, and trichloroethylene. The plot of binding energies between ethylbenzene, toluene, 1,3-dibutadiene, and trichloroethylene and the mutated pMMOs are shown in Figure 7 and the values of the binding energies of the same are recorded in Table S3. The binding energies for 1,3-dibutadiene, and trichloroethylene remained almost the same when docked with the mutants of the key interacting amino acid residues as shown in Figure 7c,d. Therefore, the study was performed considering the interaction between the ethylbenzene and toluene.

**Figure 8:**
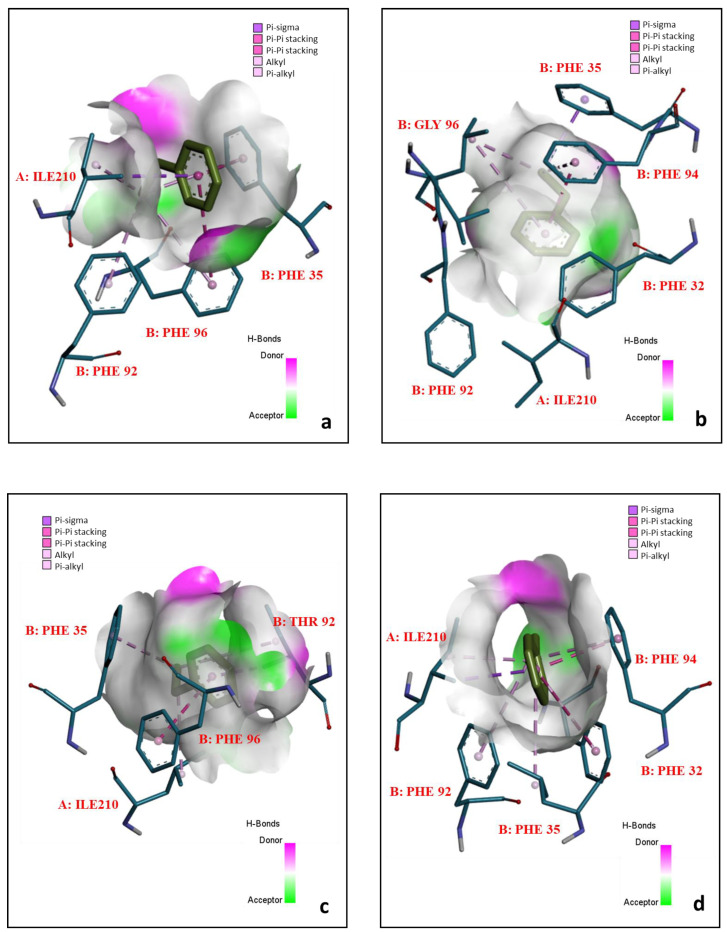

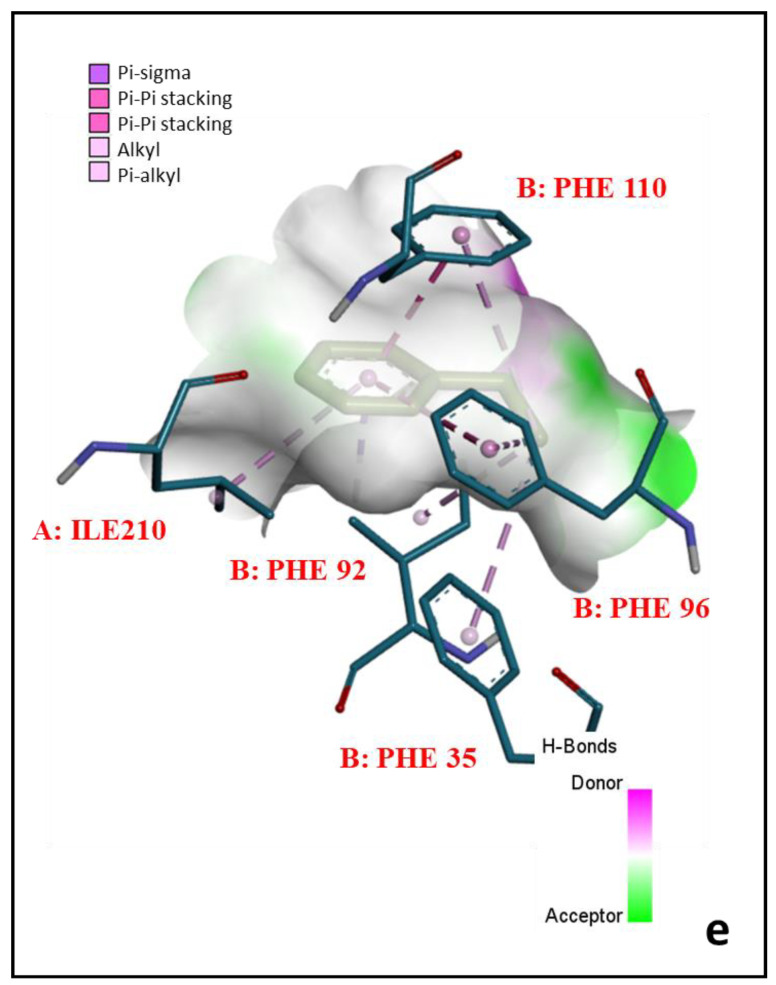
The interactions between ethylbenzene and (**a**) B:Leu31Ser, (**b**) B:Phe96Gly, (**c**) B:Phe92Thr, (**d**) B:Trp106Ala, and (**e**) B:Tyr110Phe.

While scanning for hydrogen bonds (h-bonds) within 4 Å in wild type (WT) B:Leu31, there is a presence of h-bonds with N-terminal B:Trp28, N-terminal B:Asp27, and N-terminal B:Asp27 at, 3.19, and 3.14 Å, respectively (Figure S4a). B:Leu34 and B:Leu33 are hydrogen bonded to each other at 2.79 Å. The hydrogen bonds between the amino acid residues remained intact when B:Leu31 was mutated with serine, and the hydrogen bond distances were insignificantly different as compared to the wild type (shown in Figure S5a). Among the nineteen mutants of B:Leu31, the mutant B:Leu31Ile showed the higher binding energies with ethyl benzene and toluene (−6.3 and −6.2 kcal/mol, respectively), as compared to WT B:Leu31 (ethyl benzene: −5.2 kcal/mol; toluene: −5.7 kcal/mol), as shown in Figure 7a,b. However, the percentile rank of B:Leu31Ser in terms of strain energy was 52.63% (26.20 kcal/mol). The lower the strain energy of the rotamer, the more is the stability of the protein. B:Leu31Ser showed the lowest strain energy (19.85 kcal/mol), and the subsequent highest binding energy (ethyl benzene: −6.3 kcal/mol; toluene: −6 kcal/mol). However, there were other potential candidates such as alanine, arginine, cysteine, proline, and threonine, which showed equal binding energies with ethyl benzene (−6.3 kcal/mol) but these were not promising in the case of binding energies with toluene. The PDBSUM database showed that the B:Leu31 participates in the right helical turn. The WT pMMO had the B:Leu31 as 3(10) alpha-helix (length: 35.67 Å, with 3.70 residues per turn) within the allowed region of the Ramachandran plot, which generally occurs close to the upper right of the helical region, and indicates lower stability. But serine was observed to be within the favorable region, and the analysis of the torsion angles of the rotamer of serine suggested that there were no clashes of the serine rotamer with the secondary backbone of the protein due to steric hindrances. Moreover, the promiscuity of the enzyme remained unaltered due to insignificant increase in the number of polar residues (serine) over the non-polar within the 3 Å vicinity of the pocket site 1, which allows better conformational changes and kinetics during the reaction. Figure S5a and Figure 8a shows the hydrogen bonds of B:Leu31Ser (mutant) and the interaction of B:Leu31Ser with ethylbenzene respectively. Toluene interacted in the same manner in terms of bonds and amino acid residues, and therefore the interaction of toluene with the mutants are not shown. These results indicate that B:Leu31Ser single mutation is a suitable candidate for the enhancement of catalytic activity.

The presence of two hydrogen bonds between B:Phe96 and N-terminal B:Phe92 at distances 2.43, and 2.94 Å, respectively, is shown in Figure S4b. The hydrogen bonds between the key residue with the other residues remained unchanged when B:Phe96 was mutated with glycine. From a total of nineteen mutants, the B:Phe96Gly mutant showed higher binding affinity towards ethylbenzene and toluene (−6.7, and −6.2 kcal/mol, respectively) whereas the WT B:Phe96 showed −5.2 kcal/mol, and −5.7 kcal/mol, respectively (Figure 7a,b). Furthermore, both phenylalanine and glycine are hydrophobic non-polar in nature, and hydrophobicity therefore remained unaltered within the pocket site 1. The Ramachandran plot suggested that the WT phenylalanine at the α-subunit lies within the favored region, however the rotamer lies within the allowed region. The mutant B:Phe96Gly was observed to be within the favored region of the Ramachandran plot and no steric hindrances were observed. No strain energy data were found in the databases because glycine does not have multiple rotamers. The torsion angles of the glycine were found to suitable to hold the right-handed alpha helix in position. Figure S5b and Figure 8b show the hydrogen bonds of B:Phe96 with neighbor sidechain residues and the interaction with ethylbenzene, respectively. Similarly, from the binding energy (ethyl benzene: −6.3 kcal/mol; toluene: −6 kcal/mol), strain energy (34.98 kcal/mol; percentile rank: 26.32), and favorable region in the plot, B:Phe92Thr may be a good candidate for enhancing methane oxidation, as well as B:Phe96Gly. Figure S5c and Figure 8c show the hydrogen bonds of B:Phe92 with neighbor sidechain residues and the interaction with ethylbenzene, respectively.

The binding energies of the nineteen mutants of B:Trp106 with the ethylbenzene and toluene are shown in the plots of Figure 7a,b, respectively. B:Trp106 is present within the alpha-helix (length: 24.17 Å with 3.66 residue per turn) of the enzyme, whereas B:Tyr110 is not involved in helixes or turns. When B:Trp106 and B:Tyr110 were mutated with the rest of the nineteen amino acid residues, the binding energies between the mutants and ethylbenzene remained the same (−6.5 kcal/mol), whereas the highest binding energies (−6.4 kcal/mol) were observed between B:Trp106Ala, B:Tyr110Leu and B:Tyr110Phe when docked with toluene. There were mutants (B:Trp106Phe, B:Trp106Pro, and B:Trp106Val) whose binding energies with toluene were observed to be the same (−6.4 kcal/mol). The strain energies for the B:Trp106Phe, B:Trp106Pro, and B:Trp106Val were 37.83, 87.07, and 44.24, respectively, which are considered to be too high. Besides, B:Trp106Phe, B:Trp106Pro, and B:Trp106Val were observed to affect the alpha helices of the protein and were present within the outliers of the Ramachandran plot, despite the fact that alanine is a hydrophobic non-polar amino acid and did not alter the hydrophobicity and the alpha helices. The WT amino acid, tryptophan and the rotamer were present at the outliers of the Ramachandran plot, whereas the substitute (alanine) lied within the allowable region of the plot. Similarly, B:Tyr110 was found at the outlier, while the rotamer was at the allowable region of the plot. When B:Tyr110 was mutated with leucine and phenylalanine, the binding energies between the mutated proteins and the toluene were the same (−6.4 kcal/mol). The strain energies of B:Tyr110Leu and B:Tyr110Phe were 31.14, and 25.01, respectively. Therefore, B:Tyr110Phe, with the highest binding energy, lowest strain energy and no steric clashes; and B:Trp106Ala, with promising features towards the stability of the protein backbone; are two mutants that may be suitable candidates for enhancement of methane oxidation. The interactions of these mutants are shown in Figure 8d,e.

The Ramachandran plots analyzed for the suitable mutants (B:Leu31Ser, B:Phe96Gly, B:Phe92Thr, B:Trp106Ala, and B:Tyr110Phe) describe the changes in stereochemical properties due to the substitution of amino acid residues. The Ramachandran statistics and RMSD of each mutant are shown in Table 2. The percentages of residues in the favorable region of the mutants did not change significantly (within 0.015 Å) when compared to wild-type modeled pMMO. This indicates that there may not be any changes in stereochemical properties in the pMMO due to these mutations, since the percentage of residues in the favorable region in wild type and mutants pMMO remain the same.

The mutant B:Leu31Ser resulted in gaining pyrrolidine carboxylic acid (PrrCA) level at Q6 which altered metal binding with probabilistic scores of 0.31 and 0.28, respectively. These metal bindings with probabilistic scores were calculated using a MutPhred. PrrCA at Q6 is an important factor to predict the behavior of the ion channels in transmembrane subunits; the loss of which may cause the collapse of the cell. The B-factor of a protein structure measures the atomic fluctuation profile due to thermal agitation. The gain in B-factor at B:Phe96Gly, and B:Phe92Thr shows that atoms of histidine will easily be thermally agitated at moderate temperature. The mutant, B:Trp106Ala and B:Tyr110Phe, resulted in the gain at the catalytic site at nearby amino acid residues and simultaneously loss at allosteric site at side chain residues, which did not alter metal binding. Therefore, B:Leu31Ser, B:Phe96Gly, B:Phe92Thr, B:Trp106Ala, and B:Tyr110Phe may increase the affinity of methane to interact with the active site, which could be validated experimentally. We are currently performing wet lab experiments to create mutants of the identified 8 amino acids using site-directed mutagenesis approaches.

## 4. Discussion

The existence of active sites and the overall metal content within the crystal structure of pMMO from various methanotrophs is quite controversial [20,62]. The spectroscopic and crystallographic studies suggested that the metal content within the pMMO subunits may be the permutations or the individual contributions from mononuclear copper, dinuclear copper, trinuclear copper, di-iron, and/or zinc [63]. The crystal structure of pMMO (PDB #3CHX) of *M. trichosporium* OB3b, studied earlier, using X-ray diffraction crystallography with a resolution of 3.90 Å, shows copper centers in its mononucleated and denucleated form. A similar observation has been reported for the pMMO crystal structure of *Methylococcus capsulatus* BATH which contains a trinuclear copper center and zinc center unlike *M. trichosporium* OB3b [9]. Furthermore, the poor resolution of the 3CHX structure and improper stereochemical torsions between the rotamers of amino acid residues reveals no information about its active sites. But the question arises whether these copper centers are responsible for the active site. Several literature articles contradict the notion that the active site of pMMO may be present in any of the three subunits. In one study, it was claimed that pMMO contains only mononuclear copper centers, of which one is present within the soluble α-subunit and the other within the membrane bound γ-subunit [50]. In this study, using an in-silico approach, we modeled the monomeric structure of pMMO with an attempt to correct the stereochemical torsions of the residues and thereby, to provide insight on the active sites within the pMMO of *M. trichosporium* OB3b.

Further studies related to the active sites demanded proper stereochemistry of the pMMO. Therefore, the improvements in the stereochemical behavior of the structure from 48.86% to 82.34% (favorable region) were achieved using modeling of the monomeric structure of the holoenzyme. The binding energy between the holo form of the modeled structure and the substrates revealed the interacting key residues in the PS1 (B:Trp106, B:Tyr110, A:Met203, B:Phe35, B:Ile210, B:Leu31, B:Phe32, and B:Phe96), and in other pocket sites (PS2, PS3, and PS4). This indicates that both α-subunit and β-subunit are combinedly contributing towards the PS1, and all the subunits of pMMO are contributing to the other pocket sites. Therefore, through this study, we contradict the assertion that the active site of pMMO in OB3b is present in a single subunit.

Next, it has also been reported that the substrate binding cavity of pMMO from *M. trichosporium* OB3b expresses high enantioselectivity for n-butane and n-pentane, and the binding site is close to the metal center but not perfectly graved within the center [64]. However, in our predictions, interactions were not observed at the vicinity of the either the mononuclear copper center or dinuclear copper center, although copper remains the critical cofactor towards methane oxidation. Unlike other pocket sites, PS1 was promising to accommodate all the substrates with higher binding energies (ligand RMSD: 0.00). These findings suggest that pocket site 1 may contribute towards the active site of the monomeric pMMO, and further investigations based on hydrophobicity, tunnel length, and cavity volume made in this study confirm this hypothesis. The hydrophobicity analysis and the conserved domain analysis showed that the key interacted residues in PS1 were non-polar hydrophobic, except tyrosine, and are conserved throughout the six methanotrophic genera chosen. The tunnel length and the cavity volume for pocket site 1 were observed to be 20 Å, and 3892 Å^3^, respectively, which are comparatively higher than the volume of the substrates (as calculated approximately from the radii, <1000 Å^3^), thus signifying the possible active diffusion of substrates, and therefore influence both substrate specificity and catalytic mechanism [65]. Thus, the PS1 can be inferred as one of the active sites which may solely contribute towards methane oxidation.

Very few molecular studies related to the alteration and mutation of the pMMO genes have been reported to date because tailoring the transmembrane region leads to loss of structural integrity of the cell, loss of activity during extraction and purification, and distortion of the diffusional path for methane [66]. In addition to in vivo limitations, there are considerable molecular in-vitro challenges, such as the slow growth of methanotrophs on plates during selection and non-specific reliable assay methods during validation [66]. Recent advances have revealed molecular genetics techniques to tailor the monooxygenase enzyme in methanotrophs but they are limited to sMMO [26,67]. Methane monooxygenases have a more significant role in the bioremediation of industrial wastes (such as in-situ degradation of trichloroethylene) and commercial gas to liquid conversion (which are economical as compared to Fischer-Tropsch reactions). Using methane as a substrate, along with the implementation of small-scale and large-scale reactors, methane monooxygenases are being employed to synthesize biofuels such as methanol, and other value-added products such as single-cell protein (SCP), biopolymers, pigments, organic acids, ectoine, farnesene, and vitamin B12 [68]. However, the methane oxidation rates in the wild-type *M. trichosporium* OB3b is very low (800 nmoles min^−1^) as reported, due to poor enzyme activity (<10,000 nmol min^−1^ mg^−1^), and thus limits its application to commercial fields which garner higher profits [69]. On the other hand, using both in-silico and in-vitro experiments, the literature has shown that mutagenesis within the catalytic domain of sMMO enhances the methane oxidation rates [70,71]. Therefore, with an attempt to enhance the methane uptake rate in *M. trichosporium* OB3b, using in-silico approach we mutated the key interacting residues in the PS1 with nineteen other residues and screened the mutants on the basis of strain energy, hydrophobicity, hydrogen bonding, and binding energies. We illustrated five key amino acid mutation within the α-subunit namely, B:Leu31Ser, B:Phe96Gly, B:Phe92Thr, B:Trp106Ala and B:Tyr110Phe, that would likely increase the rate of methane uptake.

In addition to validating the above results in wet lab using site-directed mutagenesis and knockout studies, the follow up paper we will report the in-vitro enhancement of methane oxidation using higher concentrations of copper, which will provide insight into the biochemical approach to this enhancement.

## 5. Conclusions

There are still several intriguing questions about methanotrophs and their key MMO enzymes. A wide range of methodologies derived from the disciplines of microbiology, enzymology, structural biology, and quantum chemistry have contributed to the search for answers to some of these questions. The literature suggests that the individual active sites are in either *pmoA* or *pmoB*, but the present study showed that at least 4 active sites are present within three subunits. The docking studies showed that the interactions between the substrate and protein are higher when multiple (two or more) subunits are working together towards interaction. Thus, all the subunits of pMMO are important for the catalysis of methane. The mutagenesis study was targeted to 8 amino acids that were majorly interacting with all the substrates. The mutagenesis study showed 5 site directed mutations, namely B:Leu31Ser, B:Phe96Gly, B:Phe92Thr, B:Trp106Ala and B:Tyr110Phe, are significantly promising for enhancing the rate of methane catalysis. Our approach towards the investigations of location of active site and further investigation of mutations to increase the catalysis rate of methane will help us in the near future to carry out the experiments in the wet lab. Gene knock down and gene silencing techniques could be used to confirm the active sites in pMMO, and site directed mutagenesis strategy could be employed to create the mutant library of the above mutants to enhance the catalysis rate of methane.

## Figures and Tables

**Table 1:** The chemical IDs of the natural substrates and nonnatural substrates used in the study.

Type of Substrates	Ligand Name	PubChem ID	Geometrical Properties		Stereometry
			Topological Polar Surface Area	Mean Square Radius (~Calculated)	Number of Atoms That Are Stereometrically Changeable
Methane homologs	Ethylbenzene	CID #7500	0 Å^2^	8.018 Å	1
	Toluene	CID #1140	0 Å^2^	7.622 Å	0
Synthetic homologs	1.3-dibutadiene	CAS #106-99-0	NA	4.34 Å	1 (tautomer)
	Trichloroethylene	CID #6575	0 Å^2^	4.89 Å	0

NA: not available.

**Table 2:** UNIPROT IDs of *pmoA*, *pmoB*, and *pmoC* of the listed methanotrophs.

Methanotrophs	Proteobacteria	UNIPROT IDs		
		*pmoA*	*pmoB*	*pmoC*
*Methylosinus trichosporium*	α-proteobacteria	Q50541	Q9KX50	Q9KX51
*Methylocystis* sp. JTC3	α-proteobacteria	W6DH29	W6D960	W6D653
*Methylococcus capsulatus* BATH	γ-proteobacteria	Q607G3	G1UBD1	Q603F1
*Methylomicrobium alcaliphilum* DSM 19304	γ-proteobacteria	G4SZ63	G4SZ64	G4SZ62
*Candidatus Methylomirabilis oxyfera*	Candidate division NC 10	D5MJE2	D5MJE0	D5MINO
*Methylacidiphilum infernorum* isolate V4	Verrucomicrobia	A9QPE3	A9QPE4	A9QPE2
*Methylocystis* sp. *strain* M	α-proteobacteria	O06122	Q9KX36	Q9KX37
*Methylocystis* sp. (strain SC2)	α-proteobacteria	Q70EF3	Q70EF2	Q70EF4
*Methylotuvimicrobium japanense*	γ-proteobacteria	Q25BV3	Q25BV2	Q25BV4
*Rhodococcus* sp. WAY2	γ-proteobacteria	A0A6P1IDD3	A0A6P1IF66	A0A6P1IAM1
*Methylovulum miyakonense*	γ-proteobacteria	E1CBX0	E1CBX1	E1CBW9
*Methylothermus subterraneus*	γ-proteobacteria	D1MYY0	D1MYY1	D1MYX9
*Methylocaldum* sp. T-025	γ-proteobacteria	A4PDX7	A4PDX8	A4PDX6

**Table 3:** Summary results of the null hypothesis tests.

Parameters of the Tests	Null Hypothesis Tests			
	f-Test		t-Test	
Mean (M)	M1: −0.033	M2: −0.032	M1: −0.033	M2: −0.032
Variance (V)	V1: 0.0000655	V2: 0.0000615	V1: 0.0000659	V2: 0.0000618
Df	690		1515	
F, T critical one-tail	1.127		1.645	
F, T critical two-tail	NA		1.962	
Coefficient (α)	0.005		0.005	
F-stat	1.065		NA	
*p*-value, one-tail	0.193		1.48539 × 10^−5^	
*p*-value, two-tail	NA		2.97079 × 10^−5^	

NA: not applicable.

**Table 4:** Comparison in Ramachandran statistics between the modeled structure and the mutants of pMMO.

Protein Names	Ramachandran Statistics		Protein Geometry				RMSD
	Favorable Region	Outlier Region	Favored Rotamers	Poor Rotamers	Bad Angles (%)	Bad Bonds (%)	
pMMO (modeled)	620 atoms (82.34%)	36 atoms (4.78%)	538 atoms (85.40%)	32 atoms (5.08%)	1.99	0.32	0.34 (with template)
Template (3CHX)	365 atoms (48.86%)	171 atoms (22.89%)	397 atoms (63.02%)	113 atoms (17.94%)	0.27	0.00	0 (with template)
B:Leu31Ser	620 atoms (82.34%)	36 atoms (4.78%)	538 atoms (85.40%)	32 atoms (5.08%)	1.99	0.32	0.05 (with the modeled wild type)
B:Phe96Gly	620 atoms (82.34%)	36 atoms (4.78%)	538 atoms (85.40%)	32 atoms (5.08%)	1.99	0.32	0.05 (with the modeled wild type)
B:Phe92Thr	620 atoms (82.34%)	36 atoms (4.78%)	538 atoms (85.40%)	32 atoms (5.08%)	1.99	0.32	0.05 (with the modeled wild type)
B:Trp106Ala	620 atoms (82.34%)	36 atoms (4.78%)	538 atoms (85.40%)	32 atoms (5.08%)	1.99	0.32	0.05 (with the modeled wild type)
B:Tyr110Phe	620 atoms (82.34%)	36 atoms (4.78%)	538 atoms (85.40%)	32 atoms (5.08%)	1.99	0.32	0.05 (with the modeled wild type)

## Data Availability

Study did not report any data.

## Supplementary Materials

The following supporting information can be downloaded at https://www.mdpi.com/article/10.3390/biom12040560/s1, Figure S1: The radius of one molecule of (a) ethylbenzene, (b) toluene, (c) 1,3-dibutadiene, and (d) trichloroethylene, Figure S2: Phylogenetic dendrograms between the 13 methanotrophs, each for pmoA, pmoB, and pmoC, individually, Figure S3: Ramachandran plots showing the stereochemical features of (a) the template structure and (b) the modeled structure. The magenta color circle represents the poor geometry of the right-handed alpha helix within the template (3CHX) and the red color circle represents the improved geometry of the region within the modeled structure, and (c) the superimposition of the template and the modeled structure. The template and the modeled structure are shown in pink and green, respectively, Figure S4: Hydrogen bond interactions of the wild-type residues (a) B:Leu31, (b) B:Phe96, (c) B:Phe92, (d) B:Trp106, and (e) B:Tyr110 with their neighbor residues. The wild-type residues are marked in sky-blue color, the neighbor residues are marked in red color, and the hydrogen bonds are shown in green color, Figure S5: Hydrogen bond interactions of the mutants (a) B:Leu31Ser, (b) B:Phe96Gly, (c) B:Phe92Thr, (d) B:Trp106Ala, and (e) B:Tyr110Phe, with their neighbor residues. The mutant residues are marked in sky-blue, the neighbor residues are marked in red, and the hydrogen bonds are shown in green, Table S1: (a) Percentage identity table of *pmoA* from the top six methanotrophs in the list used to analyze the conserved residues of the pocket sites, (b) percentage identity table of *pmoB* from the top six methanotrophs in the list used to analyze the conserved residues of the pocket sites, (c) percentage identity table of *pmoC* from the top six methanotrophs in the list used to analyze the conserved residues of the pocket sites, Table S2: Strain energies of the rotamer of the mutated amino acid residues, Table S3: Binding energies (kcal/mol) between the mutated (and wild-type) pMMO and the substrates.
