# Supplementary material for: Enhancement of Methane Catalysis Rates in Methylosinus trichosporium OB3b
Source: Biomolecules. 2022 Apr 9;12(4):560. doi: 10.3390/biom12040560 (PMC9024549; doi:10.3390/biom12040560)
Supplement: Supplementary file 1 [file biomolecules-12-00560-s001.zip › biomolecules-1627204-supplementary figures.pdf]

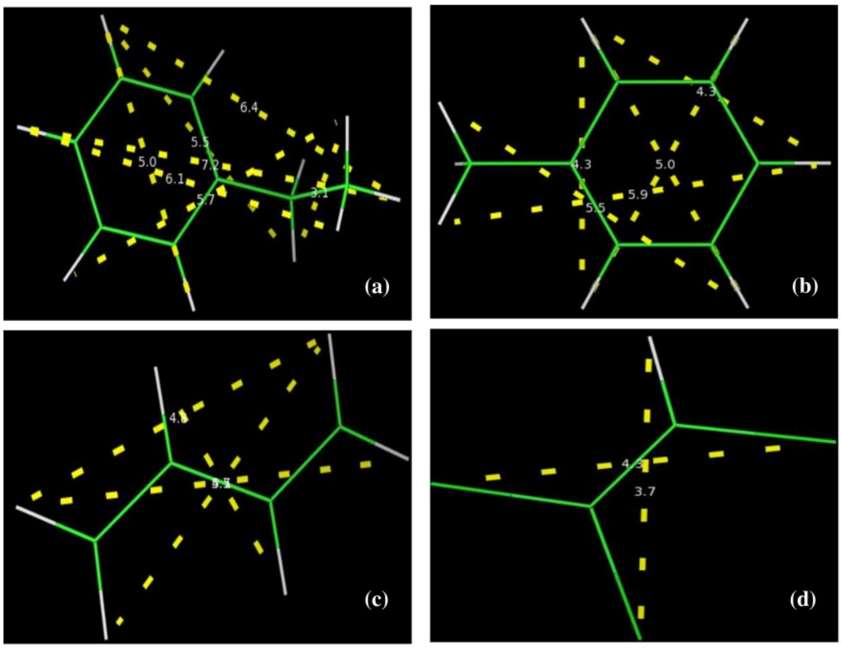

Figure S1. The radius of one molecule of (**a**) ethylbenzene, (**b**) toluene, (**c**) 1,3-dibutadiene, and (**d**) trichloroethylene.

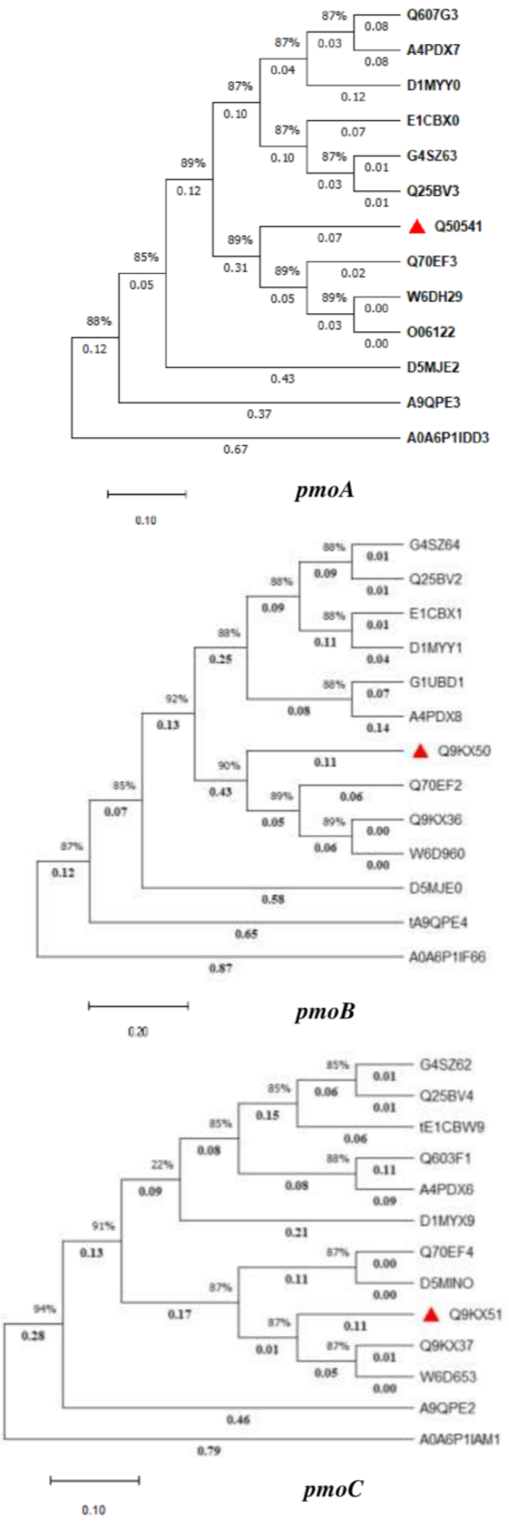

Figure S2. Phylogenetic dendrograms between the 13 methanotrophs, each for pmoA, pmoB, and pmoC, individually.

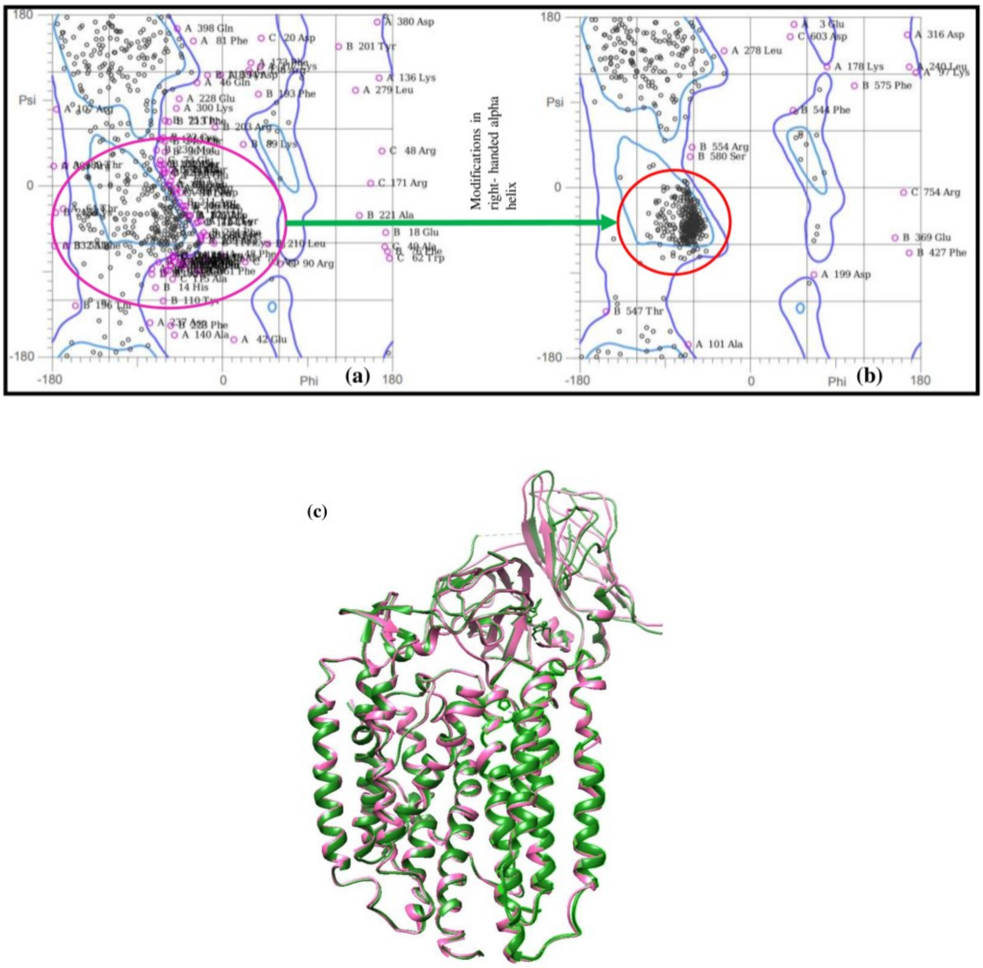

Figure S3. Ramachandran plots showing the stereochemical features of (**a**) the template structure and (**b**) the modeled structure. The magenta color circle represents the poor geometry of the right-handed alpha helix within the template (3CHX) and the red color circle represents the improved geometry of the region within the modeled structure, and (**c**) the superimposition of the template and the modeled structure. The template and the modeled structure are shown in pink and green,

respectively.

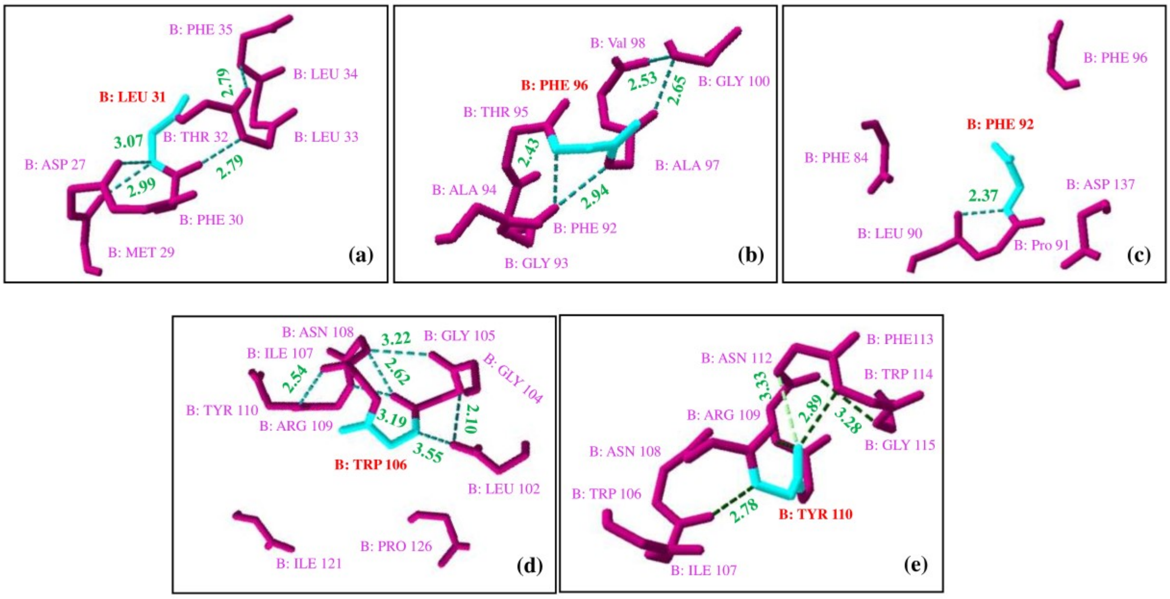

Figure S4. Hydrogen bond interactions of the wild-type residues (**a**) B:Leu31, (**b**) B:Phe96, (**c**) B:Phe92, (**d**) B:Trp106, and (**e**) B:Tyr110 with their neighbor residues. The wild-type residues are marked in sky-blue color, the neighbor residues are marked in red color, and the hydrogen bonds are shown in green color.

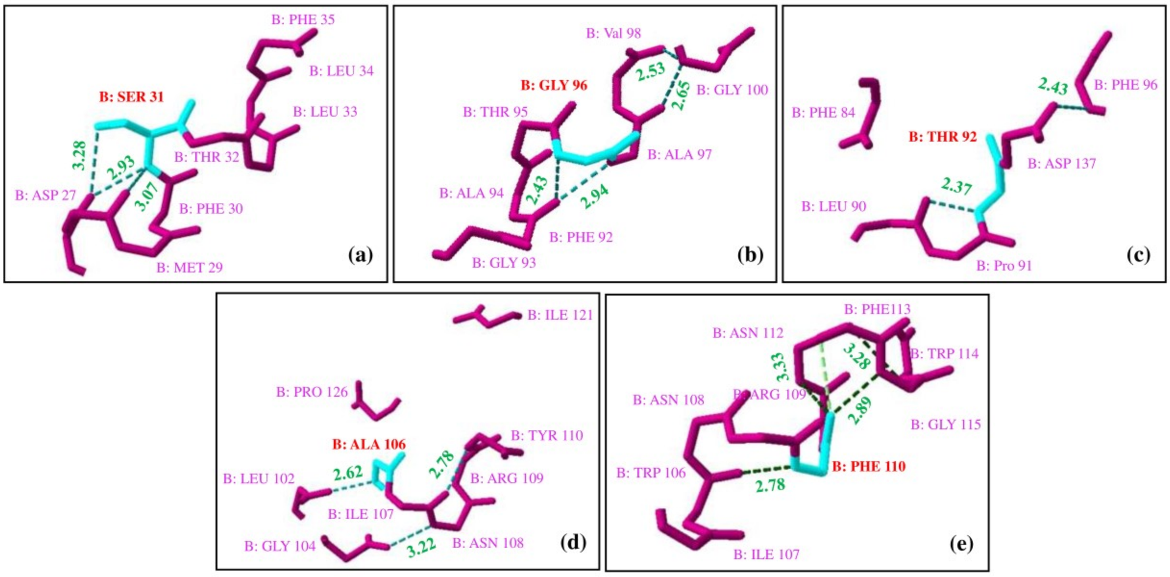

Figure S5. Hydrogen bond interactions of the mutants (**a**) B:Leu31Ser, (**b**) B:Phe96Gly, (**c**) B:Phe92Thr, (**d**) B:Trp106Ala, and (**e**) B:Tyr110Phe, with their neighbor residues. The mutant residues are marked in sky-blue, the neighbor residues are marked in red, and the hydrogen bonds are shown in green.
