# Supplementary material for: Enhancement of Methane Catalysis Rates in Methylosinus trichosporium OB3b
Source: Biomolecules. 2022 Apr 9;12(4):560. doi: 10.3390/biom12040560 (PMC9024549; doi:10.3390/biom12040560)
Supplement: Supplementary file 1 [file biomolecules-12-00560-s001.zip › Table S1.pdf]

Table S1(a): Percentage Identity table of *pmoA* from six methanotrophs used to analyze the conserved residues of the pocket sites.

UNIPROT ID	D5MJE0	A9QPE3	W6DH29	Q50541	Q607G3	G4SZ63
**D5MJE0**	100.00	49.17	49.17	47.11	53.72	50.83
**A9QPE3**	49.17	100.00	49.80	50.20	51.42	56.68
**W6DH29**	49.17	49.80	100.00	86.51	57.89	60.32
**Q50541**	47.11	50.20	86.51	100.00	58.70	59.51
**Q607G3**	53.72	51.42	57.89	58.70	100.00	78.14
**G4SZ63**	50.83	56.68	60.32	59.51	78.14	100.00

Table S1(b): Percentage Identity table of *pmoB* from six methanotrophs used to analyze the conserved residues of the pocket sites.

UNIPROT ID	A9QPE4	D5MJE0	W6D960	Q9KX50	G1UBD1	G4SZ64
**A9QPE4**	100.00	38.23	34.32	33.33	36.86	36.36
**D5MJE0**	38.23	100.00	35.96	36.12	41.56	38.54
**W6D960**	34.32	35.96	100.00	80.43	46.57	43.87
**Q9KX50**	33.33	36.12	80.43	100.00	45.52	44.31
**G1UBD1**	36.86	41.56	46.57	45.52	100.00	71.74
**G4SZ64**	36.36	38.54	43.87	44.31	71.74	100.00

Table S1(c): Percentage Identity table of *pmoC* from six methanotrophs used to analyze the conserved residues of the pocket sites.

UNIPROT ID	D5MIN0	A9QPE2	Q9KX51	W6D653	Q603F1	G4SZ62

**D5MIN0**	100.00	51.37	49.00	50.60	48.43	47.60
**A9QPE2**	51.37	100.00	53.33	51.76	50.77	49.80
**Q9KX51**	49.00	53.33	100.00	86.33	59.84	58.30
**W6D653**	50.60	51.76	86.33	100.00	60.24	59.11
**Q603F1**	48.43	50.77	59.84	60.24	100.00	72.69
**G4SZ62**	47.60	49.80	58.30	59.11	72.69	100.00
